# Icariin Alleviates Diabetes‐Associated Cognitive Dysfunction Through Modulation of LCN2–MEK/ERK Signaling‐Associated Neuroinflammation

**DOI:** 10.1002/cns.71008

**Published:** 2026-07-06

**Authors:** Xinyi Jiao, Yutong Ren, Ziman Yu, Junxiong Zhou, Danyang Wang, Bin Yan, Guoqing Tian

**Affiliations:** ^1^ Department of Traditional Chinese Medicine, Peking Union Medical College Hospital Chinese Academy of Medical Sciences & Peking Union Medical College Beijing China; ^2^ Department of Neurosurgery, Peking Union Medical College Hospital Chinese Academy of Medical Sciences & Peking Union Medical College Beijing China

**Keywords:** diabetes‐associated cognitive dysfunction, icariin, neuroinflammation

## Abstract

**Objective:**

Diabetes‐associated cognitive dysfunction (DACD) is a severe neurological complication of diabetes, yet effective preventive or therapeutic strategies remain limited. Icariin (ICA), a dietary‐derived natural flavonoid, has suggested potential neuroprotective properties in other diseases. However, its specific effects and underlying mechanisms in DACD are not fully elucidated. This study aimed to investigate the protective effects of ICA in DACD and to clarify its multi‐target mechanisms involving neuroinflammatory signaling.

**Methods:**

We explored the differentially expressed proteins between DACD and diabetes mellitus without cognitive dysfunction (DM‐noCD) patients through proteomics and validated them by ELISA. We adopted an integrated research strategy combining in vivo and in vitro experiments. In vivo, db/db diabetic mice were orally administered ICA for 4 weeks. Cognitive function was evaluated using behavioral tests, hippocampal neuroinflammation was assessed by immunofluorescence and measurement of inflammatory cytokine levels, and the regulatory effect of ICA on the LCN2‐MEK/ERK signaling pathway was evaluated through molecular biological methods. In vitro, high glucose‐stimulated HT22 hippocampal neuronal cells were utilized to validate the role of the key LCN2‐MEK/ERK pathway via LCN2 knockdown experiments.

**Results:**

ICA treatment significantly improved spatial learning and memory deficits in db/db mice. It alleviated hippocampal neuroinflammation, significantly downregulated hippocampal LCN2 expression, and inhibited phosphorylation of the MEK/ERK pathway. In HT22 cells, high glucose stimulation increased LCN2 expression and activated the MEK/ERK pathway, exacerbating inflammatory responses; ICA treatment counteracted these effects. Moreover, LCN2 knockdown suppressed MEK/ERK pathway activation, and ICA treatment induced no further changes under these conditions, suggesting that the inhibitory effect of ICA on this pathway is dependent on the presence of LCN2.

**Conclusion:**

This study suggests that ICA ameliorates DACD by targeting the LCN2‐MEK/ERK signaling pathway while alleviating neuroinflammation. These findings highlight the protective effects of ICA on DACD and its potential in other neurodegenerative disorders that may be associated with metabolic dysregulation.

## Graphical abstract

Icariin protects against diabetes‐associated cognitive dysfunction by effectively inhibiting the LCN2–MEK/ERK axis and alleviating neuroinflammation. These findings demonstrate that targeting this signaling pathway with icariin may offer a promising strategy for treating cognitive impairment in diabetes.

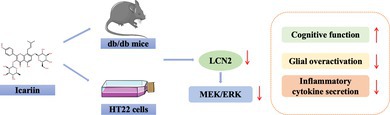

**Abbreviations**
DACD: Diabetes‐associated cognitive dysfunction
DM‐noCD: Diabetes Mellitus without Cognitive Dysfunction
ERK: Extracellular Signal‐Regulated Kinase
HE: Hematoxylin–Eosin staining
ICA: Icariin
IL‐1β: Interleukin‐1β
IL‐6: Interleukin‐6
LCN2: Lipocalin 2
MAPK: Mitogen‐Activated Protein Kinase
MEK: MAPK/ERK Kinase
MoCA: Montreal Cognitive Assessment
TNF‐α: Tumor Necrosis Factor‐α

## 1 Introduction

Type 2 Diabetes Mellitus (T2DM) has garnered increasing attention not only for its metabolic complications but also for its growing recognition of impacts on the central nervous system [1]. Epidemiological studies show that patients with T2DM have a markedly higher risk of cognitive dysfunction compared to non‐diabetic individuals [2, 3]. The growing concern of DACD, amplified by the aging population, underscores the critical urgency to investigate its underlying mechanisms [4]. Among the numerous pathogenic factors in DACD, neuroinflammation in the hippocampal region is widely recognized as a pivotal mechanism driving cognitive decline [5, 6, 7]. However, the specific molecular signaling pathways that trigger and sustain this chronic neuroinflammation remain incompletely understood.

Lipocalin‐2 (LCN2) is an acute‐phase inflammatory protein increasingly recognized for its role in neuroinflammation and cognitive disorders [8]. Elevated LCN2 levels have been observed in Alzheimer's disease and diabetic encephalopathy, where it is implicated in both glial activation and neuronal injury [9]. In STZ‐induced and high‐fat diet mouse models, upregulated hippocampal LCN2 promoted reactive gliosis, inflammatory cytokine release (e.g., IL‐6, TNF‐α), and resulted in hippocampal neuronal loss and impaired cognitive behavior [10, 11]. Circulating LCN2 levels were also positively correlated with cognitive impairment and reduced brain volume in metabolic syndrome patients [12].

Based on our previous research findings, the MAPK signaling pathway was identified as a key pathway through which icariin ameliorates DACD [13]. The MAPK family consists of three main branches: ERK, JNK, and p38, serving as an intracellular signal transduction hub that regulates critical pathophysiological processes, including inflammatory responses, apoptosis, and oxidative stress. Numerous studies have demonstrated that under diabetic and high‐glucose conditions, the JNK and p38 branches are aberrantly activated and have been confirmed to be involved in the onset and progression of DACD [14, 15]. However, the role of the MEK/ERK branch in DACD remains insufficiently studied, and its regulatory mechanisms warrant further elucidation.

Epimedium brevicornum Maxim is widely recognized as a health‐promoting food and medicinal herb in China and other East Asian countries [16, 17]. ICA, a major bioactive flavonoid derived from EM, has garnered significant attention for its anti‐inflammatory and neuroprotective properties. Pharmacokinetic studies have demonstrated that ICA and its metabolites are capable of penetrating the blood–brain barrier and reaching brain tissue, thereby establishing a critical foundation for its direct actions within the central nervous system [18, 19]. Previous studies have demonstrated that icariin can improve learning and memory abilities and alleviate cognitive dysfunction in animal models of cognitive impairment by ameliorating neuroinflammation [20, 21]. Therefore, we postulate that icariin may exert a protective effect against cognitive impairment in the context of diabetes.

This study integrates clinical plasma proteomics, db/db diabetic mouse models, and HT22 cell assays to investigate the involvement of LCN2 in DACD. We further test whether ICA administration attenuates LCN2‐mediated neuroinflammation and MEK/ERK activation, thereby improving learning and memory function. This multi‐modal experimental framework aims to elucidate the mechanistic contribution of LCN2 to DACD and evaluate ICA as a candidate therapeutic agent.

## 2 Materials and Methods

### 2.1 Human Samples

Patients with type 2 diabetes who met the inclusion criteria and were treated at the Department of Traditional Chinese Medicine, Peking Union Medical College Hospital, from July 2024 to January 2025 were enrolled as study subjects. Eight patients with DACD were randomly selected, and eight patients with DM‐noCD matched by age and sex were selected as the control group, with blood samples collected for proteomic analysis. Using the same method, another eight patients with DACD were selected and matched with DM‐noCD patients as an independent validation cohort; these selected samples were different from those used for proteomic analysis, and validation in the independent cohort was performed using ELISA. The inclusion criteria for DACD patients were as follows: (1) meeting the diagnostic criteria for type 2 diabetes according to the 2024 Chinese Guidelines for the Prevention and Treatment of Type 2 Diabetes; (2) meeting the diagnostic criteria for mild cognitive impairment according to the 2021 Expert Consensus on the Diagnosis and Treatment of Diabetes‐Related Cognitive Dysfunction (MoCA score < 26 and > 18, patient complaint or informant report of cognitive decline for ≥ 3 months, objective impairment in one or more cognitive domains (memory, executive function, attention, language, visuospatial ability, etc.), essentially normal activities of daily living, and not meeting the diagnostic criteria for dementia); (3) age 35–70 years; and (4) signed informed consent. Exclusion criteria included: Severe cardiac, hepatic, or renal insufficiency; history of psychiatric disorders or continuous use of medications affecting cognitive function for ≥ 3 months; participation in other clinical trials within the previous 3 months; pregnancy or lactation; severe hearing, visual, or language communication impairments that prevented completion of cognitive assessment; and other diseases that could cause cognitive dysfunction (e.g., cerebrovascular disease, Parkinson's disease, depression, thyroid dysfunction, etc.). Patient clinical data are presented in Tables S1 and S2. Fasting blood samples were collected from the cubital vein. Written informed consent was obtained from all participants prior to their inclusion in the study.

### 2.2 Plasma Proteomics

Plasma samples were collected using EDTA‐coated tubes and processed by the Clinical Biobank (ISO 20387) of PUMCH. Peptide separation was performed using a Vanquish Neo UHPLC system (Thermo Fisher), and mass spectrometry analysis was conducted on an Orbitrap Astral mass spectrometer (Thermo Fisher). The mass spectrometer operated in DIA mode with positive ion detection. MS1 scan range: 380–980 m/z; MS2 scan range: 150–2000 m/z. Raw data were merged and processed for identification and quantification using proprietary software. Raw data were merged and processed for protein identification and quantification using proprietary software by Meiji BioTech (China).

### 2.3 Animal Experiments

Male C57BL/6 and db/db mice (9–10 weeks old) were purchased from Jiangsu Jicui Yaokang Biotechnology Co. Ltd. (China). Mice were housed under specific pathogen‐free (SPF) conditions at 23°C ± 2°C and 50% ± 10% relative humidity on a 12‐h light/dark cycle. After 1 week of acclimatization, db/db mice were randomly divided into a model group and a treatment group (*n* = 6 per group). The treatment group received 100 mg/kg icariin dissolved in 0.5% carboxymethyl cellulose sodium (CMC‐Na) by gavage daily [22, 23, 24]; the model and control groups received the same volume of 0.5% CMC‐Na.

### 2.4 Chemical Reagents

Icariin was purchased from Yuanye Bio‐Technology Co. Ltd. (Shanghai, China). CMC‐Na was obtained from MedChemExpress (Monmouth,NJ, USA). ELISA kits were purchased from Elabscience Biotechnology (Wuhan, China). Total hippocampal RNA was extracted using TRIzol Reagent (Thermo Fisher Scientific,Waltham,MA, USA). Reverse transcription and PCR were performed using All‐in‐One First‐Strand cDNA Synthesis Kit II with dsDNase (Vazyme Biotech Co.,Nanjing, China). Real‐time PCR was carried out using SYBR Green (Vazyme Biotech Co., Nanjing, China) on a QuantStudio Real‐Time PCR System (Applied Biosystems, Thermo Fisher Scientific,Waltham,MA, USA).

### 2.5 Behavioral Testing

**Open Field Test (OFT):** Each mouse was placed in the corner of a 50 × 50 cm white box and allowed to explore freely for 5 min. Locomotor activity was recorded using an automated video tracking system. The arena was cleaned with 75% ethanol between trials.

**Y‐Maze Test:** The Y‐maze consisted of three white arms (A, B, C) arranged at 120° angles. Mice were placed in arm A and allowed to explore freely for 5 min. The sequence and frequency of arm entries were recorded. Consecutive entries into three different arms (e.g., ABC, BCA, CAB) were counted as spontaneous alternation.

**Morris Water Maze (MWM):** A circular pool (diameter: 150 cm; height: 60 cm) was filled with water to a depth of 30 cm, made opaque with non‐toxic white milk powder. Four quadrants (Q1–Q4) were designated. A hidden platform (diameter: 9 cm) was submerged 1 cm below the water surface in Q1. Training was conducted over 5 days; on the 6th day, probe trials were performed. Escape latency (max 60s), swim path, time in target quadrant, and platform crossing number were recorded.

### 2.6 Histology

Following behavioral testing, mice were euthanized, and brains were perfused with fixative and post‐fixed at 4°C for 48 h. Fixed brain tissues were dehydrated, embedded in paraffin, and sectioned into 4 μm thick coronal sections.

For histological evaluation, sections were stained with hematoxylin and eosin (HE) to assess neuronal morphology and with Nissl to evaluate neuronal survival. For immunofluorescence staining, sections were incubated overnight at 4°C with primary antibodies: Rabbit anti‐GFAP polyclonal antibody (Proteintech, 1:200) and mouse anti‐IBA1 polyclonal antibody (Proteintech, 1:200). This was followed by incubation with appropriate fluorophore‐conjugated secondary antibodies and counterstaining with DAPI. Images were acquired using a 3D HISTECH scanner and quantified by blind investigators using ImageJ software.

### 2.7 Elisa

Hippocampal homogenates and cell culture supernatants were analyzed for IL‐6, IL‐1β, and TNF‐α using commercial ELISA kits (Elabscience Biotechnology Co. Ltd, Wuhan, China). Plasma LCN2 levels in the independent validation cohort were measured using a Lianke kit.

### 2.8 Quantitative RT‐PCR

Total RNA was extracted from mouse hippocampus or cultured cells using TRIzol reagent and quantified using NanoDrop 2000. cDNA was synthesized using a reverse transcription kit (Novizan), and qPCR was performed with SYBR Green qPCR Master Mix targeting IL‐1β, TNF‐α, IL‐6, and β‐actin on a QuantStudio system. Relative mRNA levels were normalized to actin and calculated using the 2 − ΔΔCt method. Primer sequences are listed in Supplementary Table S3.

### 2.9 Western Blot

Hippocampal tissues and cells were lysed in RIPA buffer supplemented with protease and phosphatase inhibitors (NCM Biotech, China). Protein concentrations were determined using a BCA assay kit. Proteins were separated by SDS‐PAGE and transferred to PVDF membranes. After blocking, membranes were incubated with primary antibodies overnight at 4°C, washed, and then incubated with HRP‐conjugated secondary antibodies. After chemiluminescent detection, the membranes were stripped using stripping buffer and then reprobed with other primary antibodies. Detection was performed using a chemiluminescent substrate, and densitometric analysis was conducted using ImageJ software. The primary antibodies used were as follows: Anti‐Lipocalin‐2 antibody (Abcam, 1:1000), anti‐phospho‐ERK (p‐ERK) antibody (CST, 1:1000), anti‐phospho‐MEK (p‐MEK) antibody (CST, 1:1000), anti‐ERK antibody (Huabio, 1:1000), anti‐MEK antibody (CST, 1:1000), and the loading control anti‐β‐Tubulin rabbit monoclonal antibody (ABclonal, 1:5000).

### 2.10 Cell Culture and Treatment

HT22 mouse hippocampal neurons were purchased from the Cell Resource Center (Institute of Basic Medical Sciences, CAMS, Beijing, China). Cells were cultured in DMEM supplemented with 9% FBS and 1% antibiotics at 37°C with 5% CO₂. Glucose‐induced injury was modeled using 125 mM glucose for 24 h [25, 26]; the treatment group received 10 μM icariin. To exclude the osmotic effect induced by the high‐glucose environment, additional intervention groups with different concentrations of mannitol were established. The experimental results are shown in the Supplementary Figure S1. The specific protocols for cell modeling and intervention have been described in detail in our previous publication [13].

### 2.11 Lentiviral Transduction

Lentiviral vectors for LCN2 knockdown and controls were obtained from GeneChem Co. Ltd. (Shanghai, China). HT22 cells were seeded in 6‐well plates and transduced at a multiplicity of infection (MOI) of 50. After 24 h, cells were selected with puromycin at 2 μg/mL. Glucose stimulation and drug treatment were performed after successful transduction.

### 2.12 Statistical Analysis

All statistical analyses were performed using GraphPad Prism 10.0 (GraphPad Software, USA). Data are expressed as mean ± standard error of the mean (SEM). Differences between groups were assessed using unpaired *t*‐tests, one‐way ANOVA, or two‐way ANOVA, as appropriate. A *p*‐value < 0.05 was considered statistically significant.

For the proteomic data, multiple hypothesis testing was performed to control the false discovery rate (FDR). Specifically, the Benjamini‐Hochberg (BH) procedure was applied using R software (version 4.2.1, R Foundation for Statistical Computing, Austria) with the p.adjust function to adjust the *p*‐values obtained from differential expression analysis between the DACD and DM‐noCD groups. An adjusted *p*‐value (q‐value) < 0.05 was considered statistically significant.

## 3 Reaults

### 3.1 LCN2 Is Elevated in the Peripheral Blood of Diabetic Patients With Cognitive Dysfunction

**FIGURE 1:**
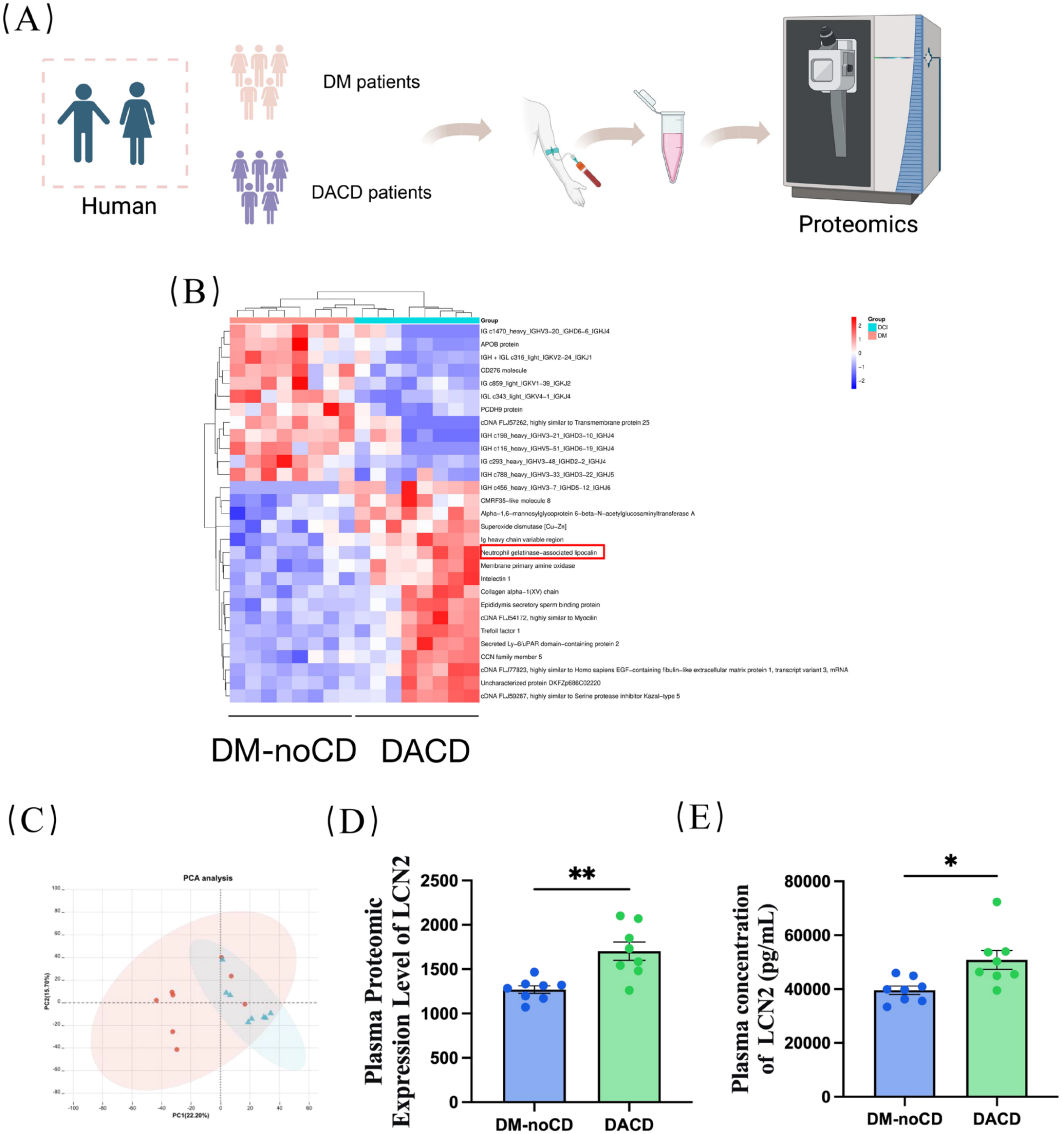
LCN2 showed significant differences between diabetic patient groups with and without cognitive impairment. (A) Proteomic Workflow of the Two Patient Groups. (B) Heatmap of differentially expressed proteins between the two groups. The protein indicated by the red box is LCN2. (C) Principal component analysis (PCA). Red represents the DACD group, and blue represents the DM‐noCD group. (D) Statistical analysis of plasma proteomic LCN2 expression between the two groups. (E) Statistical comparison of plasma LCN2 concentration between the two groups. Data are presented as mean ± SEM. *p < 0.05, **p < 0.01 vs. DM‐noCD group.

To identify proteins associated with cognitive impairment in diabetic patients, proteomic analysis was performed on peripheral blood samples from DM‐noCD and DACD (Figure 1A). A heatmap of significantly altered proteins further supported the presence of robust proteomic differences (Figure 1B). Principal component analysis (PCA) revealed a distinct separation between the two groups, indicating differential protein expression patterns (Figure 1C). Further statistical analysis of the proteomic data revealed that LCN2 was significantly upregulated in the DACD group compared to the DM‐noCD group (*p* < 0.01), suggesting its potential role in the pathophysiology of cognitive decline in diabetes (Figure 1D). Furthermore, we validated these findings in an independent cohort, confirming that plasma LCN2 concentration was significantly higher in the DACD group than in the DM‐noCD group (p < 0.05) (Figure 1E).

### 3.2 ICA Improves Learning and Memory Abilities in Db/Db Mice

**FIGURE 2:**
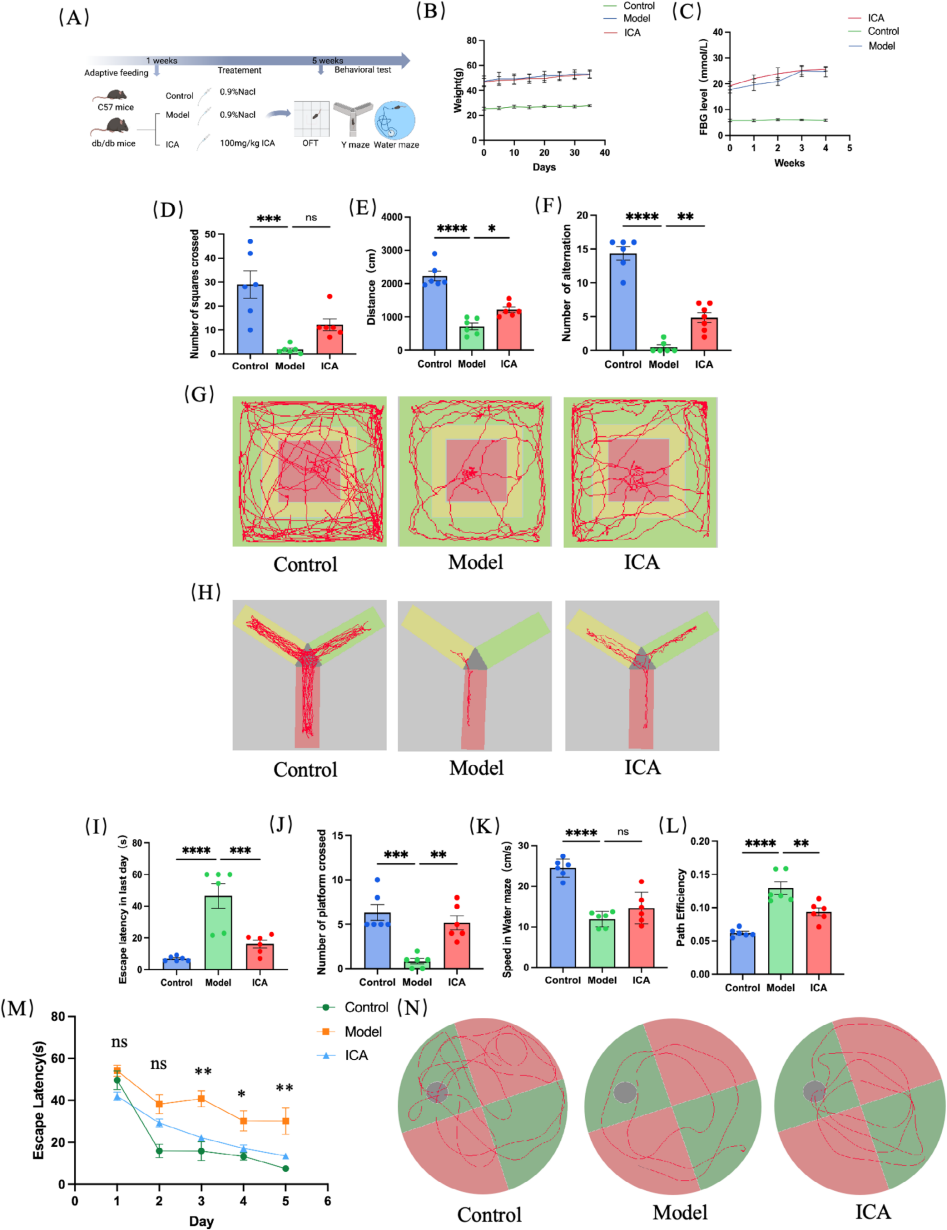
ICA Ameliorates Cognitive Function in db/db Mice. (A) Schematic diagram of the experimental design. (B) Body weight of mice. (C) Fasting Blood Glucose of mice. (D) Frequency of entries into the center zone in the OFT. (E) Total locomotor distance in the OFT. (F) Number of alternations in the Y‐maze test. (G) Representative movement traces of mice in the OFT. (H) Representative movement traces of mice in the Y‐maze test. (I) Escape latency during the probe trial in the MWM. (J) Number of platform crossings during the probe trial in the MWM. (K) Swimming speed during the probe trial in the MWM. (L) Path Efficiency in the MWM. (M) Escape latency during the training phase of the MWM. (N) Representative swimming paths of mice in the MWM. Data are presented as mean ± SEM. *N* = 6, ns, not significant, *p < 0.05, **p < 0.01, ***p < 0.001 vs. Model group.

To evaluate the behavioral effects of ICA on DACD, we conducted a series of behavioral tests, including the OFT, Y‐maze, and MWM (Figure 2A). Body weight (Figure 2B) and fasting blood glucose (Figure 2C) of mice in each group were also recorded.

In the OFT, diabetic model mice exhibited a significantly lower frequency of entries into the center zone compared to the control group (*p* < 0.001), indicating increased anxiety‐like behavior (Figure 2D). However, caution is warranted when interpreting this result, as the total locomotor distance was also significantly lower in the model group compared to the control group. Since the frequency of center entries in the open field test is influenced by both anxiety levels and locomotor activity, the observed reduction may be partially attributed to decreased motor ability rather than solely to increased anxiety‐like behavior. Although ICA treatment did not significantly increase the number of center entries, it significantly enhanced total locomotor distance compared to the model group (*p* < 0.05), suggesting that ICA may partially alleviate anxiety, improve locomotor activity, or both (Figure 2E). Representative movement traces support these findings (Figure 2G).

In the Y‐maze test, which assesses short‐term spatial working memory, the model group exhibited a significant reduction in spontaneous alternation behavior compared to the control group, while ICA treatment improved the number of alternations (Figure 2F). These results indicate that ICA enhances working memory in diabetic mice (Figure 2H).

The Morris water maze test was employed to evaluate long‐term spatial learning and memory. During the five‐day acquisition phase, escape latency progressively decreased across all groups, indicating intact basic learning ability (Figure 2M). However, in the probe trial conducted on day 6, the model group exhibited significantly prolonged escape latency (Figure 2I) and fewer platform crossings (Figure 2J) compared to the control group, suggesting marked impairment of spatial memory. ICA intervention significantly increased the number of platform crossings in model mice, indicating partial restoration of cognitive function.

Notably, due to their obese phenotype, db/db mice showed significantly reduced swimming speed compared to the control group (Figure 2K), which may confound the interpretation of time‐dependent measures such as escape latency. To exclude the interference of motor performance differences in the assessment of spatial memory, we further calculated path efficiency, defined as the ratio of the straight‐line distance from the entry point to the target platform to the actual total swimming distance (i.e., straight‐line distance/actual distance). This metric reflects the directness of spatial search strategies and is independent of absolute swimming speed [27]. The results showed that path efficiency in the model group was significantly lower than that in the control group, and ICA treatment significantly improved this metric (Figure 2L). Representative swimming paths from each group further visually illustrate these behavioral differences (Figure 2N).

### 3.3 ICA Preserves Hippocampal Neuronal Structure in Db/Db Mice

**FIGURE 3:**
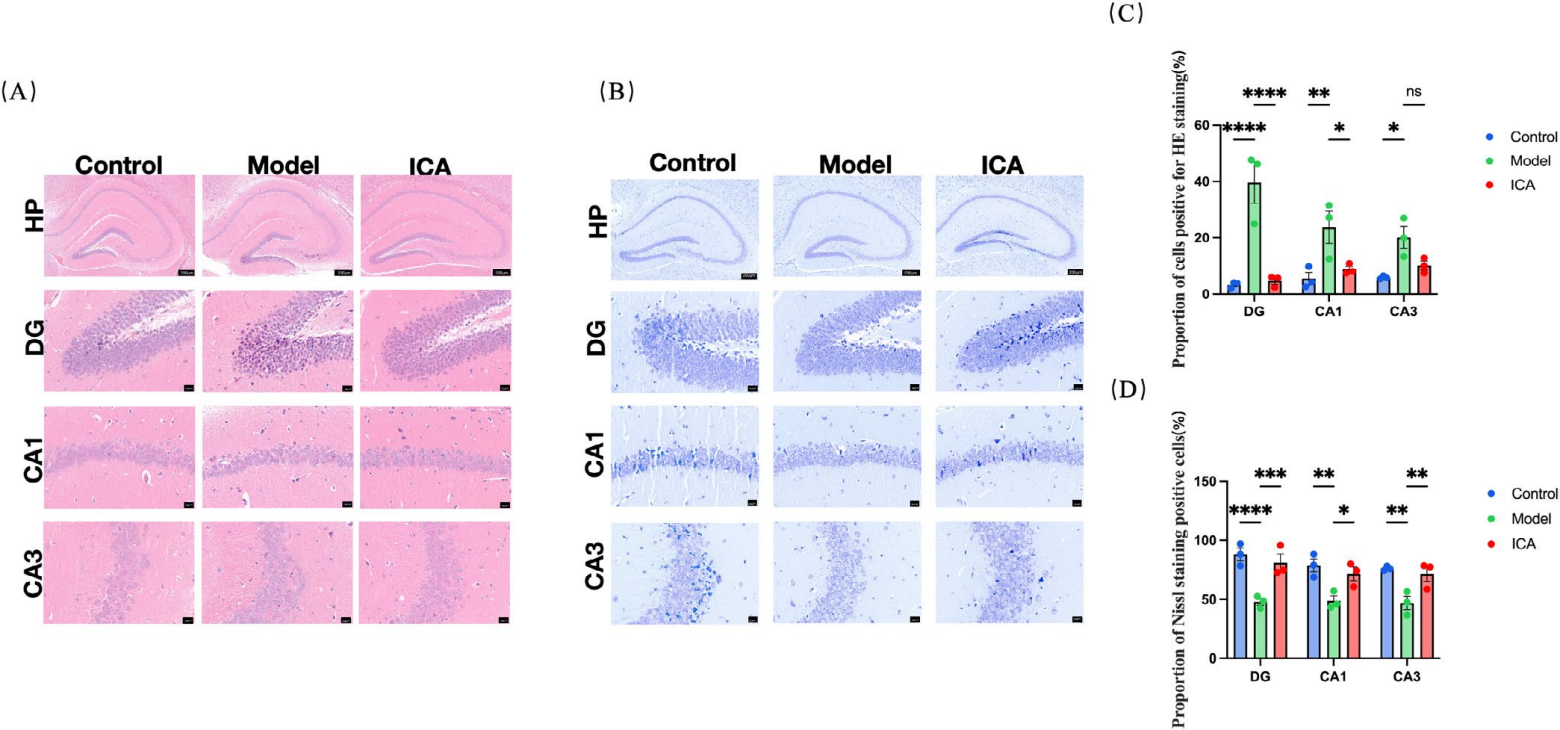
ICA ameliorates hippocampal pathology in db/db mice. (A) The HE staining results. (B) Nissl staining results. (C) Proportion of cells positive for HE staining. (D) Proportion of Nissl staining positive cells. Data are presented as mean ± SEM. *N* = 3, ns, not significant, *p < 0.05, **p < 0.01, ***p < 0.001, ****p < 0.0001 vs. Model group.

The hippocampus plays a central role in learning and memory, and structural damage in this region is commonly associated with cognitive impairment [28]. To evaluate the neuroprotective effect of ICA, HE staining and Nissl staining were performed to assess neuronal morphology in the hippocampus (Figure 3A,B).

In HE staining (Figure 3A), hippocampal neurons in the control group were arranged tightly with clear structure and plump morphology. In contrast, db/db mice showed obvious structural abnormalities, including neuronal disorganization and signs of degeneration such as cell shrinkage and necrosis. Notably, ICA‐treated mice exhibited markedly improved neuronal architecture, with a more regular arrangement and clearer boundaries, suggesting that ICA ameliorates diabetes‐induced hippocampal damage.

Nissl staining further confirmed these findings (Figure 3B). The Control group showed abundant Nissl bodies in the hippocampal neurons, indicating healthy protein synthesis activity. In the Model group, the number of Nissl bodies was substantially reduced, suggesting neuronal damage. ICA treatment partially restored Nissl body density, further supporting the neuroprotective effects of ICA in diabetic mice.

Quantitative analysis of histological changes by HE staining (Figure 3C) revealed that compared with the control group, the proportion of abnormal neurons in the DG, CA1, and CA3 regions of the hippocampus was significantly increased in the model group, characterized by pathological changes such as cell shrinkage, hyperchromatism, and nuclear pyknosis (*p* < 0.0001, *p* < 0.01, *p* < 0.05, respectively), indicating severe impairment of neuronal structural integrity under diabetic conditions. Following ICA intervention, the proportion of abnormal cells was significantly decreased in the DG and CA1 regions compared with the model group, while no significant difference was observed in the CA3 region (*p* < 0.0001, *p* < 0.05, ns), suggesting that ICA effectively alleviates diabetes‐induced hippocampal histopathological damage and improves neuronal morphology.

Quantitative analysis of the proportion of Nissl staining‐positive cells (Figure 3D) revealed that compared with the control group, the proportions of Nissl‐positive cells in the DG, CA1, and CA3 regions of the hippocampus were significantly decreased in the model group (*p* < 0.0001, *p* < 0.01, *p* < 0.01, respectively), indicating significant hippocampal neuronal damage under diabetic conditions. Compared with the model group, the ICA intervention group exhibited a significant increase in the proportion of Nissl‐positive cells in the DG, CA1, and CA3 regions (*p* < 0.001, *p* < 0.05, *p* < 0.01, respectively), suggesting that ICA effectively alleviates hippocampal neuronal damage.

These histological results provide morphological evidence that ICA protects hippocampal neurons from diabetes‐related structural damage, which may underlie its cognitive benefits observed in behavioral tests.

### 3.4 ICA Attenuates Neuroinflammation in Db/Db Mice by Inhibiting the LCN2–MEK/ERK Axis

Based on integrated proteomics and network pharmacology analyses [13], we hypothesized that the LCN2–MEK/ERK signaling pathway may be involved in ICA's neuroprotective effects in DACD. To validate this hypothesis, we examined changes in this pathway in the hippocampus using WB and immunohistochemistry (IHC).

**FIGURE 4:**
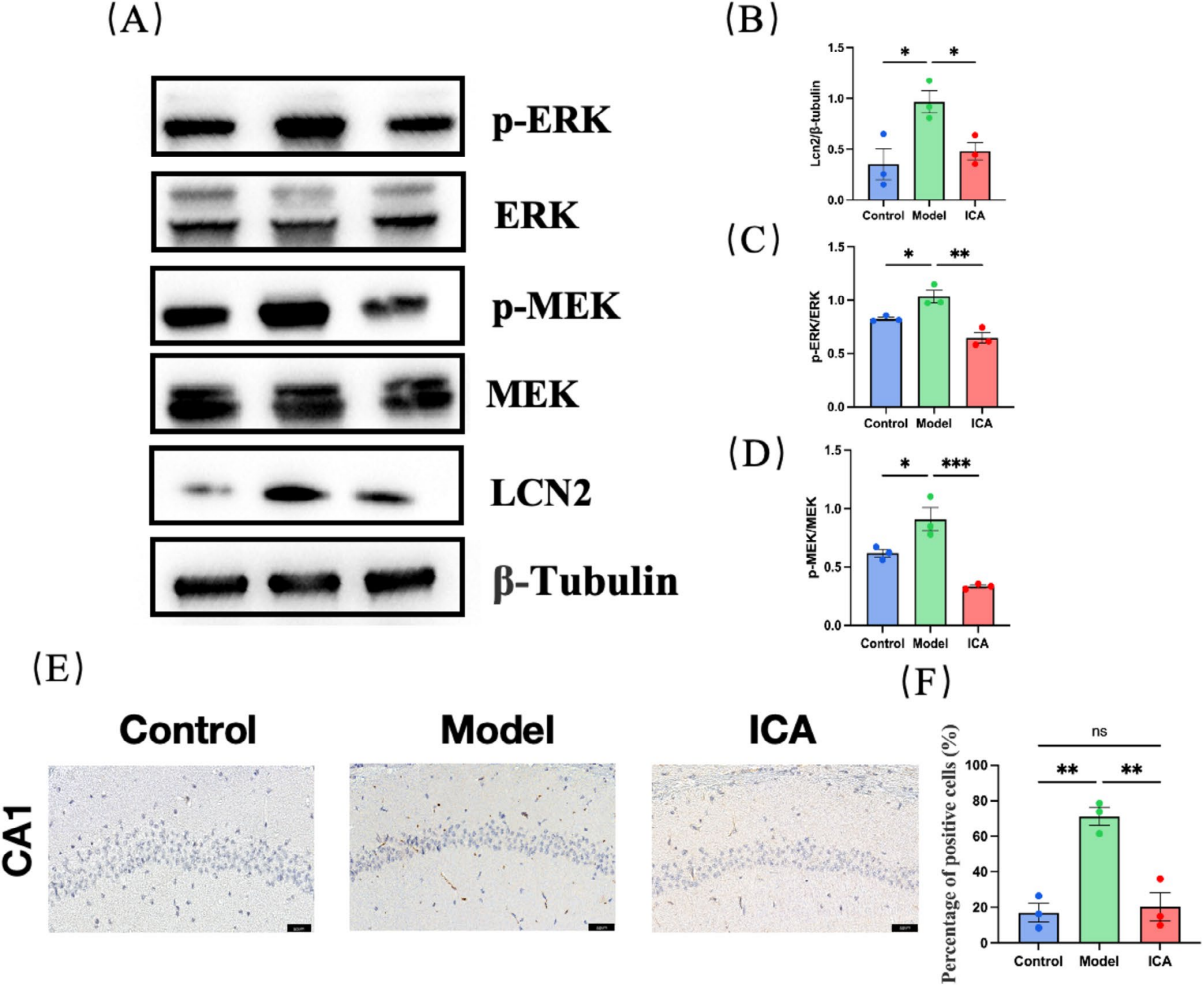
ICA suppresses LCN2‐MEK/ERK pathway‐related protein expression in db/db mice. (A) Protein expression levels of p‐ERK, ERK, p‐MEK, MEK, and LCN2 in mouse hippocampal tissues. (B) p‐ERK/ERK ratio. (C) p‐MEK/MEK ratio. (D) LCN2/β‐tubulin ratio. (E) Immunohistochemical staining of LCN2‐positive cells in the hippocampal CA1 region. (F) Quantitative analysis of LCN2‐positive cells. Data are presented as mean ± SEM. *N* = 3. ns, not significant, *p < 0.05, **p < 0.01, ***p < 0.001 vs. Model group.

Western blot analysis showed elevated expression of LCN2 protein and increased phosphorylation of MEK and ERK in the model group compared to controls. Specifically, the ratios of phosphorylated MEK to total MEK (p‐MEK/MEK) and phosphorylated ERK to total ERK (p‐ERK/ERK) were significantly higher in the model group, indicating activation of the MEK/ERK signaling cascade (Figure 4A–D). ICA treatment reversed these changes, leading to reduced LCN2 expression and suppression of MEK/ERK pathway activation.

Consistently, immunohistochemical staining of the hippocampal CA1 region revealed a marked increase in LCN2‐positive cells in the model group, which was significantly reduced following ICA treatment (Figure 4E,F).

These results provide correlational evidence consistent with the hypothesis that ICA alleviates neuroinflammation in diabetic mice by modulating the LCN2–MEK/ERK pathway, suggesting a potential involvement in reducing hippocampal inflammation and protecting neuronal function.

### 3.5 ICA Attenuates Glial Activation and Reduces Pro‐Inflammatory Cytokine Levels in the Hippocampus of Db/Db Mice

To investigate the anti‐inflammatory mechanisms of ICA in DACD, we evaluated glial activation by immunofluorescence staining of Iba1 and GFAP in the hippocampal CA1, CA3, and DG regions, and quantified the levels of pro‐inflammatory cytokines IL‐1β, IL‐6, and TNF‐α in the hippocampus using RT‐qPCR and ELISA.

**FIGURE 5:**
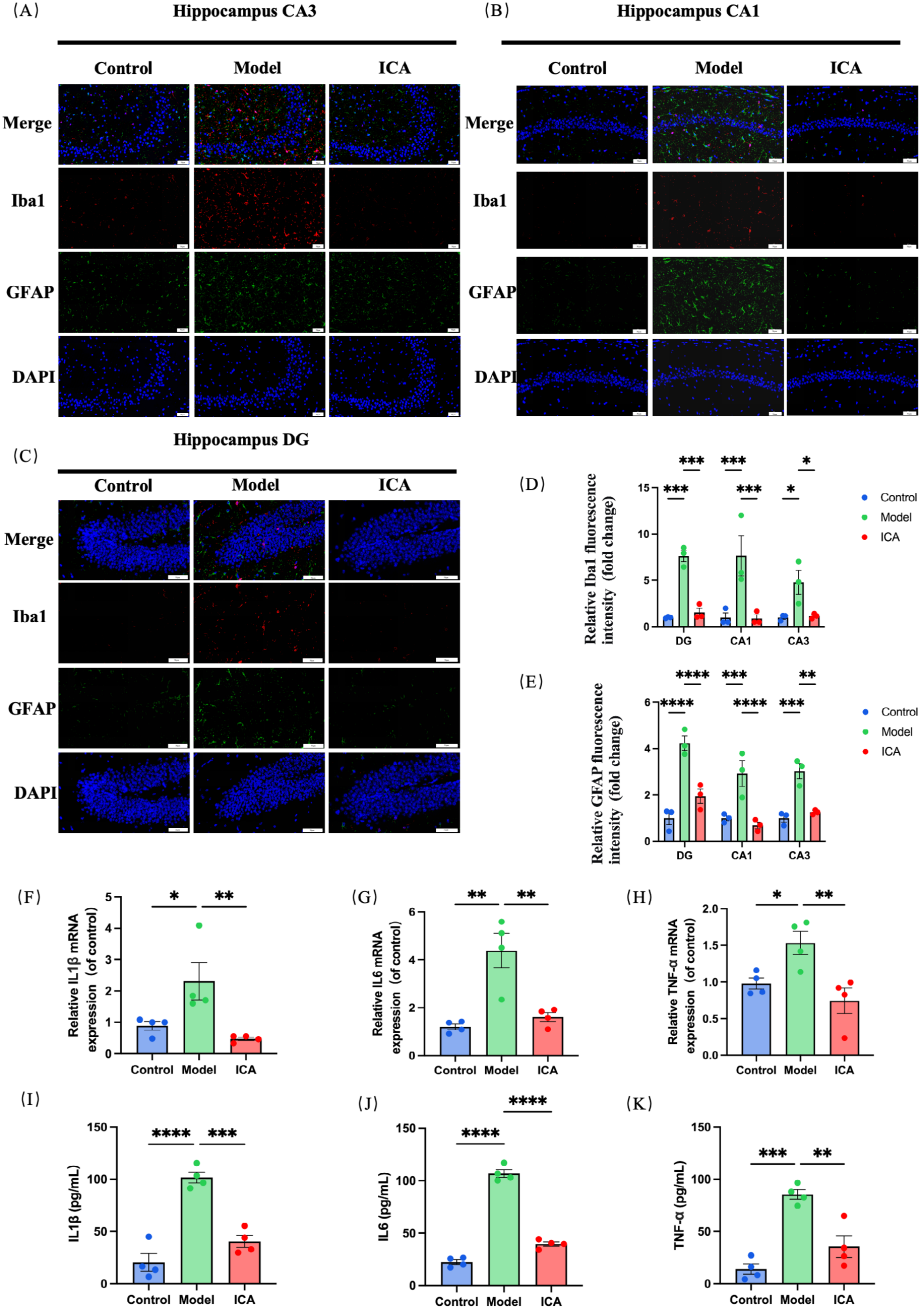
ICA alleviates neuroinflammation in db/db mice. (A–C) Representative immunofluorescence staining of Iba1 and GFAP in the hippocampal CA1, CA3, and DG regions (scale bar = 50 μm). (D) Quantitative analysis of relative fluorescence intensity of Iba1 (fold change vs. control). (E) Quantitative analysis of relative fluorescence intensity of GAFP (fold change vs. control). (F–H) mRNA expression levels of IL‐1β, IL‐6, and TNF‐α in mouse hippocampal tissues (*n* = 3). (I–K) Protein expression levels of IL‐1β, IL‐6, and TNF‐α in mouse hippocampal tissues measured by ELISA (*n* = 4). Data are presented as mean ± SEM. ns, not significant, *p < 0.05, **p < 0.01, ***p < 0.001, ****p < 0.0001 vs. Model group.

Immunofluorescence analysis revealed that the Model group exhibited significantly increased expression of GFAP and Iba1 in all three hippocampal subregions, characterized by hypertrophic astrocytes and amoeboid microglia, indicating excessive glial activation. ICA treatment markedly reduced glial activation and restored cellular morphology compared with the Model group (Figure 5A–C).

At the transcriptional level, RT‐qPCR analysis showed that mRNA expression of IL‐1β, IL‐6, and TNF‐α was significantly upregulated in the hippocampus of the Model group compared with controls (IL‐1β, *p* < 0.05; IL‐6, *p* < 0.01; TNF‐α, *p* < 0.05). ICA treatment significantly decreased the expression of all three cytokines (IL‐1β, *p* < 0.01; IL‐6, *p* < 0.01; TNF‐α, *p* < 0.01) (Figure 5F–H).

Consistently, ELISA results demonstrated that the protein levels of IL‐1β, IL‐6, and TNF‐α in hippocampal tissue were significantly elevated in the Model group compared with controls, indicating a robust central inflammatory response. Following ICA treatment, these cytokine levels were markedly reduced: IL‐1β (*p* < 0.001), IL‐6 (*p* < 0.0001), and TNF‐α (*p* < 0.01) (Figure 5I–K).

Collectively, these findings demonstrate that ICA significantly suppresses glial activation and reduces pro‐inflammatory cytokine expression at both the transcriptional and protein levels in the hippocampus, highlighting its therapeutic potential in mitigating central inflammation in DACD.

### 3.6 ICA Regulates the MEK/ERK Signaling Pathway in an LCN2‐Dependent Manner

To further investigate whether ICA regulates neuroinflammation through the LCN2–MEK/ERK signaling pathway, we performed in vitro experiments using HT22 cells exposed to 125 mM high glucose (HG). Prior to HG treatment, mannitol was used as an osmotic control to exclude osmotic effects (Supplementary Figure S1). Subsequently, the cells were either treated with ICA or subjected to LCN2 knockdown (LV‐shLCN2), and corresponding lentiviral negative control groups were established to rule out any potential interference from the lentiviral vector itself.

**FIGURE 6:**
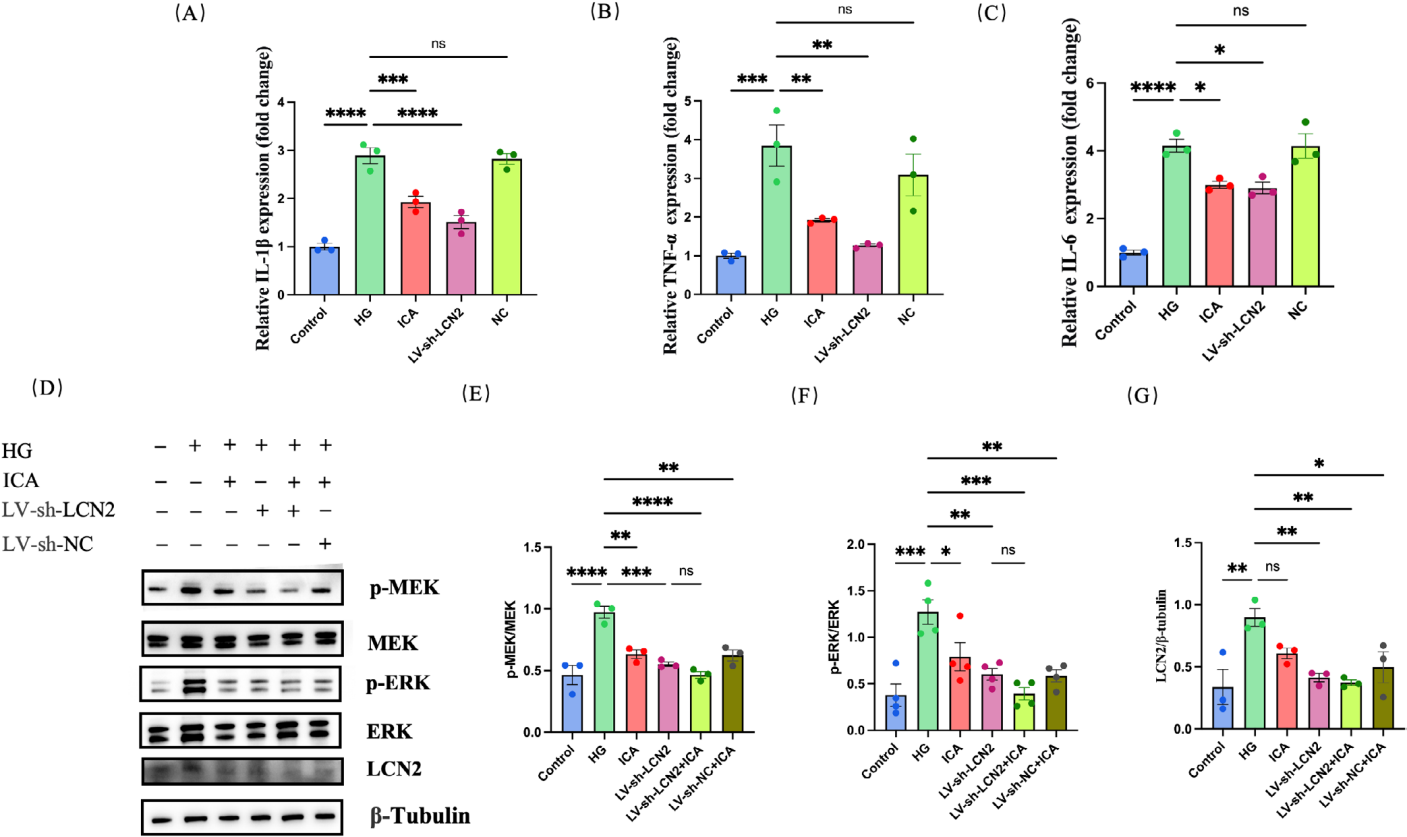
ICA Modulates Inflammatory Cytokines and the MEK/ERK Pathway via Regulation of LCN2 Expression. (A) Relative IL‐1β levels in cell culture supernatant across groups (*n* = 3). (B) Relative TNF‐α levels in cell culture supernatant across groups (*n* = 3). (C) Relative IL‐6 levels in cell culture supernatant across groups (*n* = 3). (D) Western blot analysis of p‐ERK, ERK, p‐MEK, MEK, and LCN2 protein expression with or without sh‐LCN2. (E) Quantitative analysis of p‐MEK/MEK ratios from D (*n* = 3). (F) Quantitative analysis of p‐ERK/ERK ratios from D (*n* = 4). (G) Quantitative analysis of LCN2/β‐tubulin ratios from D (*n* = 3). Data are presented as mean ± SEM. *p < 0.05, **p < 0.01, ***p < 0.001, ****p < 0.0001 vs. Model group.

ELISA results showed that HG stimulation significantly increased the secretion of the pro‐inflammatory cytokines IL‐1β, IL‐6, and TNF‐α, while both ICA treatment and LCN2 knockdown markedly reduced their levels (Figure 6A–C). These findings suggest that LCN2 is involved in HG‐induced inflammatory responses and that ICA exerts its anti‐inflammatory effects, at least in part, through modulation of LCN2.

Western blot analysis revealed that HG stimulation significantly upregulated LCN2 protein expression and activated the MEK/ERK signaling pathway, as evidenced by increased p‐MEK/MEK and p‐ERK/ERK ratios (*p* < 0.0001, *p* < 0.001), whereas ICA treatment effectively reversed these changes (*p* < 0.01, *p* < 0.05). To further validate whether ICA regulates this pathway through LCN2, we performed experiments in LCN2‐knockdown HT22 cells. We observed that LCN2 knockdown alone reduced the p‐MEK/MEK and p‐ERK/ERK ratios (*p* < 0.001, *p* < 0.05), and ICA treatment did not induce significant differences under these conditions (ns) (Figure 6E–G). These results indicate that LCN2 is an essential mediator of MEK/ERK pathway activation, and that the inhibitory effect of ICA on this pathway is dependent on the presence of LCN2.

Taken together, these findings demonstrate that ICA modulates the MEK/ERK signaling pathway through an LCN2‐dependent mechanism, thereby alleviating neuroinflammation. The anti‐inflammatory effects of ICA are at least partially dependent on the integrity of this pathway.

## 4 Discussion

A growing body of evidence suggests that ICA, a bioactive compound extracted from Epimedium, exhibits beneficial effects in various diabetic complications, including nephropathy, neuropathy, and endothelial dysfunction [29, 30, 31, 32]. However, whether ICA can improve DACD and the underlying mechanisms remains unclear. In this study, through proteomic analysis, we identified LCN2 as a candidate molecule associated with the progression from diabetes to cognitive impairment and further systematically evaluated the therapeutic potential of ICA through in vivo and in vitro experiments. Our results showed that ICA significantly improved cognitive function and alleviated neuroinflammation in diabetic mice.

In the behavioral experiments, the changes in locomotor activity observed in the open field test warrant caution when interpreting the anti‐anxiety‐like effects, as center zone exploration is inherently correlated with overall locomotor activity. Additionally, in the Y‐maze test, the total number of arm entries was not recorded; therefore, although the Y‐maze has low motor demands, we cannot completely rule out the potential influence of locomotor activity on alternation behavior. Despite these limitations, the consistent evidence of cognitive improvement in the Morris water maze test—which is less independent on swimming speed—along with the partial restoration of locomotor activity in the open field test, collectively suggests that ICA may ameliorate anxiety‐like behavior and cognitive function in diabetic mice, supporting its therapeutic potential for DACD. Future studies should further optimize the experimental design, including increasing the sample size, recording the total number of arm entries in the Y‐maze test to correct for locomotor activity, and employing more refined metrics in the open field test to better distinguish between anxiety‐like behavior and motor function effects.

The hippocampus plays a central role in spatial learning and memory. Inflammatory responses in this brain region are known to impair cognitive function [33]. In diabetic animals, increased expression of inflammatory cytokines such as IL‐1β, IL‐6, and TNF‐α has been observed in the hippocampus [34]. These peripheral and central inflammatory mediators can disrupt neuronal structure, promote synaptic dysfunction, and contribute to cognitive deficits [35, 36]. Although the role of these cytokines in diabetic neuroinflammation is well established, the upstream molecular regulators remain unclear. LCN2 is a potential candidate molecule within this upstream regulatory network. Previous studies have reported that upregulation of LCN2 is associated with blood–brain barrier disruption and the onset of neuroinflammation [37, 38]. Hyperglycemia has been identified as one of the factors stimulating LCN2 overexpression [39]. Through proteomic analysis, we observed significant upregulation of lipocalin‐2 (LCN2) in diabetic patients with cognitive impairment.

In the diabetic mouse model, we further validated this association: Hippocampal LCN2 expression was elevated in model mice, and ICA treatment significantly reduced its expression, accompanied by decreased levels of pro‐inflammatory cytokines. To further clarify the causal role of LCN2 in the mechanism of action of ICA, we performed LCN2 knockdown experiments in hippocampal neurons. The results showed that LCN2 knockdown alone was sufficient to inhibit the activation of the downstream MEK/ERK pathway and reduce the expression of inflammatory factors, and under these conditions, ICA treatment produced no additional inhibitory effect. This suggests that LCN2 is an essential molecule for ICA‐mediated regulation of the MEK/ERK pathway and its anti‐inflammatory effects. However, knockdown experiments can only demonstrate the necessity of LCN2, but not its sufficiency. To fully validate the causal role of LCN2 in the effects of ICA, future studies should further perform LCN2 overexpression experiments.

In addition to the above mechanistic findings, the translational potential of the results from this study warrants further exploration. The doses of ICA used in this study (100 mg/kg in mice, 10 μM in cells) were based on previous literature reports and effective in vitro study foundations. However, to achieve the translation from animal experiments to clinical applications, a deeper understanding of its pharmacokinetic properties is necessary. Current studies indicate that although ICA can cross the blood–brain barrier [18]， its permeability is limited [40]， and its oral bioavailability is low, as it is rapidly metabolized in vivo into products such as icariside. Therefore, whether the observed central neuroprotective effects are attributable to the parent compound or its active metabolites remains unclear, a question that directly concerns the drug's ability to penetrate the blood–brain barrier and achieve effective concentrations in brain tissue. Future studies should prioritize the use of techniques such as LC–MS/MS to determine the brain/plasma distribution ratio of ICA and its metabolites under diabetic conditions. Furthermore, given that diabetic cognitive impairment requires long‐term intervention, conducting long‐term toxicity studies and safety window assessments of ICA will provide critical data for its feasibility as a disease‐modifying therapy. Future research should progressively elucidate the above pharmacokinetic and pharmacodynamic relationships to advance ICA toward clinical application for the treatment of diabetic cognitive impairment.

However, several limitations exist in this study. First, although we employed multi‐omics approaches and enhanced the mechanistic credibility through in vitro knockdown experiments, the precise causal role of LCN2 in mediating the in vivo effects of ICA has not been fully established. Future studies should combine in vivo genetic manipulation experiments, such as LCN2 knockout mice or hippocampus‐specific knockdown/rescue experiments, to provide more direct causal evidence. Second, the safety and pharmacokinetic properties of ICA in long‐term application warrant further investigation. Third, this study primarily focused on the MEK/ERK branch of the MAPK pathway and did not examine the effects of ICA on other key MAPK pathways such as JNK and p38. Therefore, we cannot exclude the possibility that ICA exerts synergistic anti‐inflammatory effects by regulating these pathways. Fourth, this study only included patients from Peking Union Medical College Hospital. The single‐center design and relatively small sample size may have introduced selection bias and limited the generalizability of our findings. We acknowledge this limitation and suggest that future multi‐center studies with larger cohorts are needed to validate our conclusions.

## Author Contributions

**Xinyi Jiao:** conceptualization, investigation, methodology, validation, software, data curation, formal analysis, writing – original draft. **Yutong Ren:** methodology, validation. **Ziman Yu:** methodology, validation. **Junxiong Zhou:** data curation. **Danyang Wang:** data curation. **Yan:** data curation. **Guoqing Tian:** writing – review and editing, visualization, funding acquisition, project administration, resources, supervision.

## Funding

This work was supported by the National Natural Science Foundation of China (No. 82074404).

## Ethics Statement

The animal experiments involved in this study were approved by the Animal Welfare and Ethics Committee of Peking Union Medical College Hospital, Chinese Academy of Medical Sciences (Approval No. XHDW‐2024‐142) and were conducted in strict accordance with relevant guidelines. The human experimental components of this study were approved by the Ethics Committee of Peking Union Medical College Hospital, Chinese Academy of Medical Sciences (Approval No. I‐23PJ1022), and written informed consent was obtained from all participants.

## Conflicts of Interest

The authors declare no conflicts of interest.

## Supporting information


**Figure S1:** CCK‐8 assay showing cell viability of HT22 cells after 24 h treatment with different concentrations of mannitol. Glu: Glucose, Man: Mannitol. Data are presented as mean ± SEM. ns, not significant, ***p < 0.001, ****p < 0.0001 vs. Glu 25 mM group.

**Table S1:** Participant characteristics of the cohort for proteomic analysis.

**Table S2:** Participant characteristics of the independent cohort.

**Table S3:** Primer sequence.

## Data Availability

The data that support the findings of this study are available from the corresponding author, [Guoqing Tian, Email address: tz_xhtcm@163.com], upon reasonable request.
