# Supplementary material for: Icariin Alleviates Diabetes‐Associated Cognitive Dysfunction Through Modulation of LCN2–MEK/ERK Signaling‐Associated Neuroinflammation
Source: CNS Neurosci Ther. 2026 Jul 6;32(7):e71008. doi: 10.1002/cns.71008 (PMC13337538; doi:10.1002/cns.71008)
Supplement: Supplementary file 1 — Figure S1: CCK‐8 assay showing cell viability of HT22 cells after 24 h treatment with different concentrations of mannitol. Glu: Glucose, Man: Mannitol. Data are presented as mean ± SEM. ns, not significant, ***p < 0.001, ****p < 0.0001 vs. Glu 25 mM group. [file CNS-32-e71008-s004.docx]

**Supplementary Figure S1**


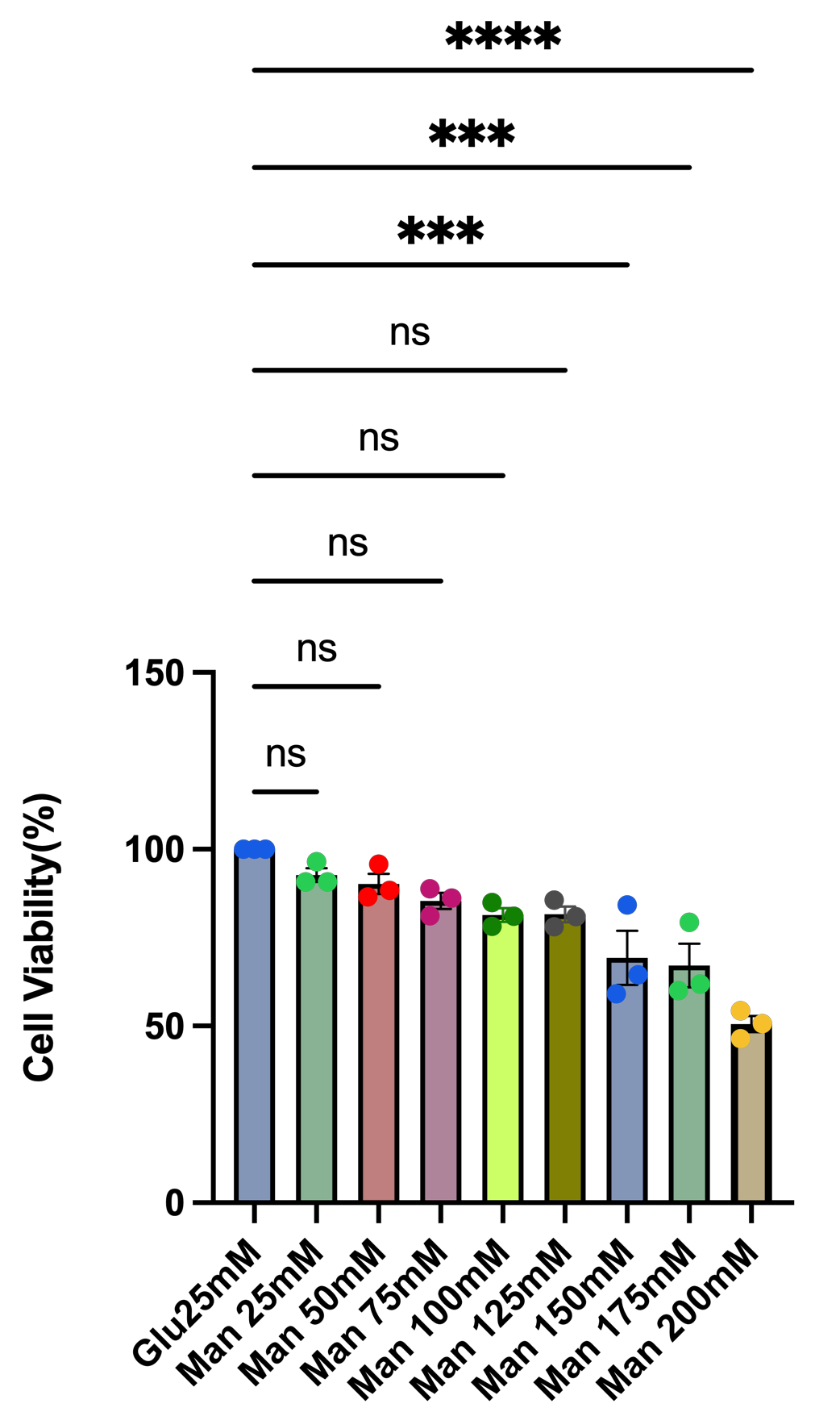


Fig S1 CCK-8 assay showing cell viability of HT22 cells after 24 h treatment with different concentrations of mannitol.Glu: Glucose, Man:mannitol. Data are presented as mean ± SEM. ns, not significant, ****p*<0.001, *****p*<0.0001 vs. Glu 25mM group.
