# Supplementary material for: Icariin Alleviates Diabetes‐Associated Cognitive Dysfunction Through Modulation of LCN2–MEK/ERK Signaling‐Associated Neuroinflammation
Source: CNS Neurosci Ther. 2026 Jul 6;32(7):e71008. doi: 10.1002/cns.71008 (PMC13337538; doi:10.1002/cns.71008)
Supplement: Supplementary file 2 — Table S1: Participant characteristics of the cohort for proteomic analysis. [file CNS-32-e71008-s002.docx]

**Supplementary Table S1. Participant characteristics of the cohort for proteomic analysis**

| Clinical characteristics | DM-noCD cases | DACD cases | *P* value |
| --- | --- | --- | --- |
| N | 8 | 8 | - |
| Sex (male) | 5 | 6 | - |
| Age (years) | 58.63±4.432 | 65±1.680 | 0.200 |
| BMI (kg/m2) | 28.44±1.941 | 24.43±0.8132 | 0.0773 |
| Duration of diabetes (years) | 11.25±3.183 | 16±3.813 | 0.3551 |
| FPG(mmol/L) | 8.74±1.097 | 7.913±0.4533 | 0.4983 |
| HbA1c (%) | 6.913±0.3613 | 7.329±0.6774 | 0.5835 |
| Serum creatinine (μmol/L) | 72.13±12.80 | 107.9±37.65 | 0.3838 |
| Total cholesterol (mmol/L) | 4.198±0.4881 | 4.213±0.4332 | 0.9820 |
| LDL cholesterol (mmol/L) | 2.529±0.3815 | 2.473±0.3435 | 0.9143 |
| HDL cholesterol (mmol/L) | 1.099±0.05992 | 1.108±0.1060 | 0.9437 |
| Triglycerides (mmol/L) | 1.369±0.1897 | 1.600±0.1682 | 0.3772 |
| HCY (μmol/L) | 12.96±1.951 | 19.76±3.789 | 0.1329 |

Notes: BMI, body mass index. HbA1c, hemoglobin A1c. LDL, low-density lipoprotein. HDL, high-density lipoprotein.. HCY, homocysteine.
