# Supplementary material for: Icariin Alleviates Diabetes‐Associated Cognitive Dysfunction Through Modulation of LCN2–MEK/ERK Signaling‐Associated Neuroinflammation
Source: CNS Neurosci Ther. 2026 Jul 6;32(7):e71008. doi: 10.1002/cns.71008 (PMC13337538; doi:10.1002/cns.71008)
Supplement: Supplementary file 3 — Table S2: Participant characteristics of the independent cohort. [file CNS-32-e71008-s001.docx]

**Supplementary Table S2. Participant characteristics of the independent cohort**

| Clinical characteristics | DM-noCD cases | DACD cases | *P* value |
| --- | --- | --- | --- |
| N | 8 | 8 | - |
| Sex (male) | 4 | 5 | - |
| Age (years) | 63.5±3.718 | 63.5±2.478 | >0.99 |
| BMI (kg/m2) | 24.33±1.088 | 23.56±1.270 | 0.6543 |
| Duration of diabetes (years) | 14±3.857 | 16.25±2.202 | 0.6397 |
| FPG(mmol/L) | 8.3±0.7419 | 7.3±1.026 | 0.4541 |
| HbA1c (%) | 7.025±0.4204 | 8.163±0.9883 | 0.3075 |
| Serum creatinine (μmol/L) | 56±5.445 | 59±4.066 | 0.6656 |
| Total cholesterol (mmol/L) | 3.955±0.1983 | 5.065±0.6467 | 0.1231 |
| LDL cholesterol (mmol/L) | 2.284±0.1471 | 2.881±0.4859 | 0.2588 |
| HDL cholesterol (mmol/L) | 1.124±0.08285 | 1.326±0.1373 | 0.2273 |
| Triglycerides (mmol/L) | 1.588±0.3935 | 2.02±0.6086 | 0.5602 |
| HCY (μmol/L) | 9.843±0.6388 | 10.69±0.6354 | 0.3676 |

Notes:BMI, body mass index. HbA1c, hemoglobin A1c. LDL, low-density lipoprotein. HDL, high-density lipoprotein.. HCY, homocysteine.
