# Supplementary material for: Icariin Alleviates Diabetes‐Associated Cognitive Dysfunction Through Modulation of LCN2–MEK/ERK Signaling‐Associated Neuroinflammation
Source: CNS Neurosci Ther. 2026 Jul 6;32(7):e71008. doi: 10.1002/cns.71008 (PMC13337538; doi:10.1002/cns.71008)
Supplement: Supplementary file 4 — Table S3: Primer sequence. [file CNS-32-e71008-s003.docx]

**Supplementary Table S3. Primer sequence**

| Primer name | Primer sequence |
| --- | --- |
| IL6-F | GCCTTCTTGGGACTGATGCT |
| IL6-R | TGTGACTCCAGCTTATCTCTTGG |
| IL1β-F | TGCCACCTTTTGACAGTGATG |
| IL1β-R | TGATGTGCTGCTGCGAGATT |
| TNF-F | TAGCCCACGTCGTAGCAAAC |
| TNF-R | TGTCTTTGAGATCCATGCCGT |
| β-ACTIN-F | CAGATGTGGATCAGCAAGCAGGA |
| β-ACTIN-R | CGCAACTAAGTCATAGTCCGCCTA |
